# Genetic diversity and antifungal susceptibility profiles in causative agents of sporotrichosis

**DOI:** 10.1186/1471-2334-14-219

**Published:** 2014-04-23

**Authors:** Anderson Messias Rodrigues, G Sybren de Hoog, Débora de Cássia Pires, Raimunda Sâmia Nogueira Brihante, José Júlio da Costa Sidrim, Marcos Fabio Gadelha, Arnaldo Lopes Colombo, Zoilo Pires de Camargo

**Affiliations:** 1 Department of Microbiology, Immunology and Parasitology, Cellular Biology Division, Federal University of São Paulo (UNIFESP), São Paulo, SP, Brazil; 2 Centraalbureau voor Schimmelcultures, KNAW Fungal Biodiversity Centre, Utrecht, The Netherlands; 3 Department of Medicine, Infectious Diseases Section, Federal University of São Paulo (UNIFESP), São Paulo, SP, Brazil; 4 Specialized Medical Mycology Center, Postgraduate Program in Medical Microbiology, Federal University of Ceará, Fortaleza, Ceará, Brazil; 5 Postgraduate Program in Veterinary Science, State University of Ceará, Fortaleza, Ceará, Brazil

**Keywords:** *Sporothrix schenckii*, *Sporothrix brasiliensis*, Multidrug resistance, MIC, MFC, Intraspecific diversity

## Abstract

**Background:**

Sporotrichosis is a chronic subcutaneous mycosis of humans and animals, which is typically acquired by traumatic inoculation of plant material contaminated with *Sporothrix* propagules, or via animals, mainly felines. *Sporothrix* infections notably occur in outbreaks, with large epidemics currently taking place in southeastern Brazil and northeastern China. Pathogenic species include *Sporothrix brasiliensis*, *Sporothrix schenckii s. str.*, *Sporothrix globosa*, and *Sporothrix luriei*, which exhibit differing geographical distribution, virulence, and resistance to antifungals. The phylogenetically remote species *Sporothrix mexicana* also shows a mild pathogenic potential*.*

**Methods:**

We assessed a genetically diverse panel of 68 strains. Susceptibility profiles of medically important *Sporothrix* species were evaluated by measuring the MICs and MFCs for amphotericin B (AMB), fluconazole (FLC), itraconazole (ITC), voriconazole (VRC), posaconazole (PCZ), flucytosine (5FC), and caspofungin (CAS). Haplotype networks were constructed to reveal interspecific divergences within clinical *Sporothrix* species to evaluate genetically deviant isolates.

**Results:**

ITC and PCZ were moderately effective against *S. brasiliensis* (MIC_90_ = 2 and 2 μg/mL, respectively) and *S. schenckii* (MIC_90_ = 4 and 2 μg/mL, respectively). PCZ also showed low MICs against the rare species *S. mexicana*. 5FC, CAS, and FLC showed no antifungal activity against any *Sporothrix* species. The minimum fungicidal concentration ranged from 2 to >16 μg/mL for AMB against *S. brasiliensis* and *S. schenckii*, while the MFC_90_ was >16 μg/mL for ITC, VRC, and PCZ.

**Conclusion:**

*Sporothrix* species in general showed high degrees of resistance against antifungals. Evaluating a genetically diverse panel of strains revealed evidence of multidrug resistant phenotypes, underlining the need for molecular identification of etiologic agents to predict therapeutic outcome.

## Background

Sporotrichosis is a (sub)cutaneous mycosis of man and animals that is highly prevalent in tropical and subtropical areas, and currently reaching epidemic proportions in Brazil [1-3] and China [4,5]. Over the last decade, sporotrichosis has changed from a relatively obscure endemic infection to an epidemic zoonotic health problem in the South and Southeast Regions of Brazil [2]. Infection typically occurs by cutaneous inoculation of the microorganism from environmental sources [6,7] or via zoonotic transmission [3]. Sporotrichosis is a chronic disease that can spread lymphatically and most often presents as a localized lesion with subcutaneous nodules that eventually break through the skin [7,8]. Although rare, hematogenous dissemination can also occur depending on the immune status of the host [9].

Recent studies on molecular phylogeny clarified species boundaries within clinical isolates of *Sporothrix* and provided strong support for many cryptic groups. The major causative agents of sporotrichosis are several closely related thermodimorphic fungi of the genus *Sporothrix *[10,11], including *Sporothrix brasiliensis*, *Sporothrix schenckii s. str.*, *Sporothrix globosa*, and *Sporothrix luriei *[2,5]. These species have distinct virulence profiles [12], karyotypes [13] and geographic distributions ranging from regional [1,10] to global [5]. The *Sporothrix* genus, embedded in the plant-associated order Ophiostomatales, exhibits high genetic diversity [10,11], which is accompanied by a diversity of *in vitro* responses to the main antifungal agents [14] used in sporotrichosis treatment [7,15]. Such agents include potassium iodide [16] and itraconazole [17] for localized cutaneous and lymphocutaneous forms, and amphotericin B for disseminated cases [6,9,16]. Screening for antifungal susceptibilities during epidemiological surveillance programs could help to uncover putative multidrug resistant *Sporothrix* strains, and improve our ability to adjust therapeutic regimens and reduce relapse.

Despite the considerable spread of the Brazilian epidemic of sporotrichosis over the last two decades [1-3,10] and the increased occurrence of atypical cases [9], no study of this illness to date has integrated molecular epidemiology and antifungal drug susceptibility. The present study aimed to evaluate the resistance profiles to currently used antifungals in a genetically diverse panel of *Sporothrix* strains representing the main pathogenic species.

## Methods

### Fungal isolates

This study included 68 *Sporothrix* isolates that were obtained from clinical lesions of patients with varying degrees of disease severity (n = 65 human/n = 2 animal) or from environmental sources (n = 1). The isolates were originally received as *S. schenckii s.l*., and were subsequently identified down to the molecular species level by Rodrigues *et al.*[1,3,18] and Fernandes *et al. *[12,19] (Additional file 1: Table S1). Isolates were stored at room temperature in slant cultures on Sabouraud dextrose agar (SDA; Difco Laboratories, Detroit, MI). Ethical approval was provided by Institutional Committee (UNIFESP-0244/11).

### Antifungal agents and susceptibility testing

Culture conditions and inoculum preparation were as described by Marimon *et al. *[14]. Using the methods recommended in the CLSI standard document M38-A2, we evaluated minimum inhibitory concentrations (MIC) for the following seven antifungal agents: amphotericin B (AMB), fluconazole (FLC), itraconazole (ITC), voriconazole (VRC), posaconazole (PCZ), flucytosine (5FC), and caspofungin (CAS). The final drug concentrations ranged from 0.03 to 16 μg/mL for AMB, ITC, VRC, PCZ, and CAS, and from 0.12 to 64 μg/mL for 5FC and FLC. The microplates were incubated at 35°C and read after 72 h. *Candida parapsilosis* ATCC 22019 and *Candida krusei* ATCC 6258 were used as quality controls for the experiments.

To obtain the minimum fungicidal concentration (MFC), for each serial dilution, 10 μL was taken from each well with no visible growth and spread on Sabouraud dextrose agar (Difco Laboratories, USA). Plates were then incubated at 28°C for 48–72 h. The MFC was defined as the lowest drug concentration that yielded three or fewer colonies, i.e., 95.5 to 99% killing activity [20]. The Mann–Whitney U test was used to determine the distribution of MIC and MFC data. A *p* value of <0.05 was considered to indicate statistical significance.

### Molecular characterization

The isolates were genetically characterized [1,10] based on calmodulin gene sequencing using the primers CL1 (5′-GAR TWC AAG GAG GCC TTC TC) and CL2A (5′-TTT TTG CAT CAT GAG TTG GAC) [21], as well as sequencing part of the rRNA operon [5] using the primers ITS1 (5′-TCC GTA GGT GAA CCT TGC GG) and ITS4 (5′-TCC TCC GCT TAT TGA TAT GC) [22]. We choose these loci because they have previously been extensively used for phylogeny and taxonomy of this complex of cryptic species [1-3,5,10,11,23]. To increase the quality of sequence data, sense and antisense sequences were determined with an ABI 3730 DNA Analyser (Applied Biosystems, Inc., Foster City, CA, USA) and thereafter assembled into single sequences via CAP3 using bases with a *Phred* quality score of ≥30. All sequences were deposited online at GenBank (Additional file 1: Table S1).

Phylogenetic histories were reconstructed using maximum likelihood (ML) and neighbor-joining (NJ) methods with the Mega5 software [24], using 1,000 bootstrap replicates to estimate the confidence values for individual branches [25]. Evolutionary distances were calculated using the Tamura 3-parameter model [26]. The rate of change between sites was modeled with a gamma distribution (shape parameter = 1). Nucleotide (π) and haplotype (Hd) diversities [27] were estimated using DnaSP software version 5.10 [28]. Haplotype networks were used to visualize differences and diversity among *S. brasiliensis* and *S. schenckii* sequence data [28]. Gaps and missing data were excluded in the calculations. Median-joining networks [29] for the concatenate dataset (*CAL* + ITS) were obtained and visualized using the Network 4.610 software, as previously described by Rodrigues *et al. *[3].

## Results

**Figure 1:**
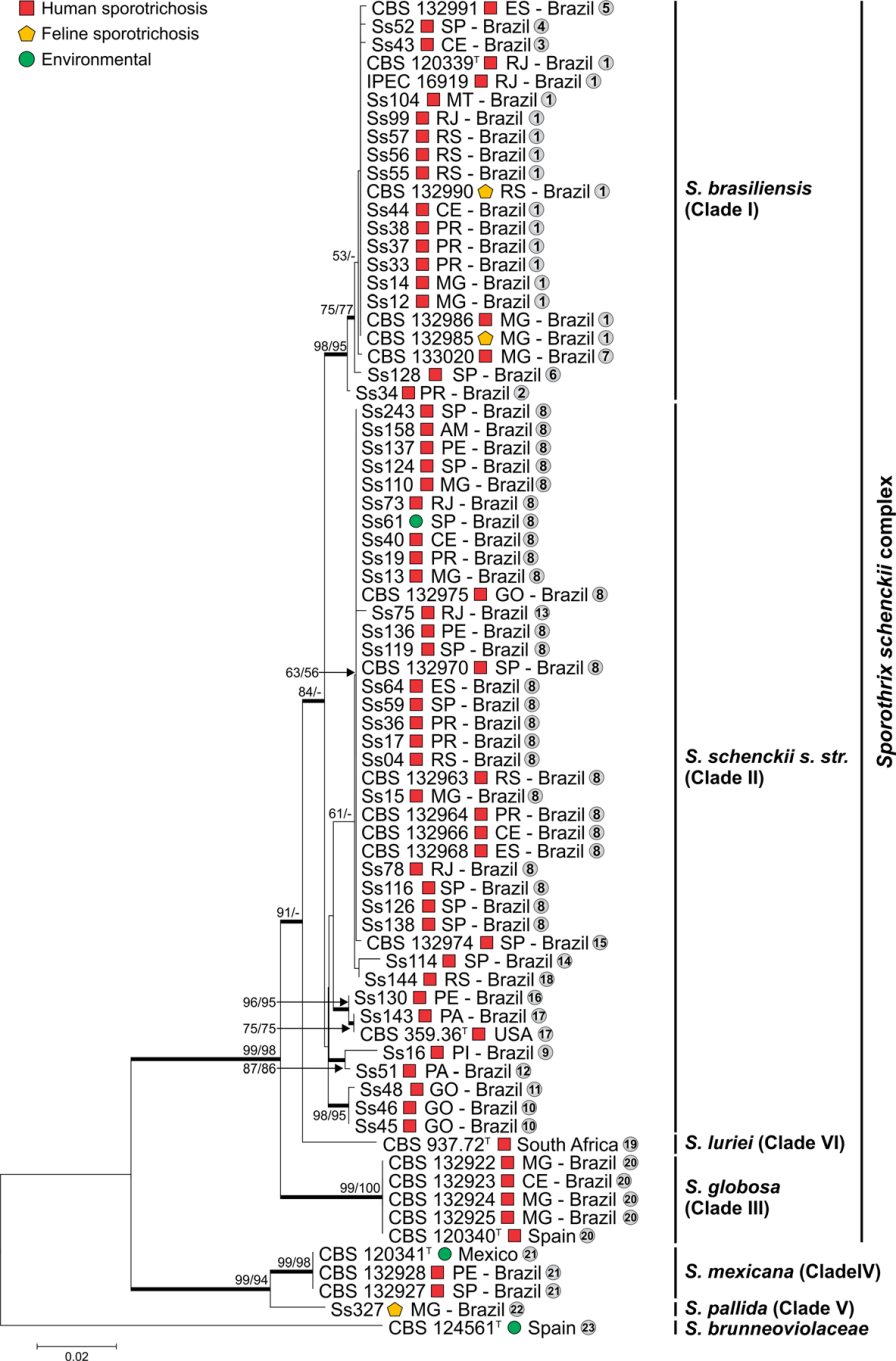
**Phylogenetic relationships of calmodulin-encoding gene and ITS sequences among clinical and environmental *Sporothrix *species.** The phylogenetic tree were estimated in MEGA5 using the Tamura 3-parameter model. The numbers close to the branches represent indices of support (NJ/ML) based on 1,000 bootstrap replications. The branches with bootstrap support higher than 70% are indicated in bold. Haplotypes numbers were assigned to each isolate and are indicated inside the circles.

The combined dataset (*CAL* + ITS) yielded a sequence alignment of 1,397 positions, including 866 invariable characters, 232 variable parsimony-informative sites (16.6%), and 185 variable singletons. The topologies of the ML and NJ trees were congruent (*I*_*cong*_ = 3.66; *p* = 1.68 e-24) [30] and the isolates were distributed among the six major clades (Figure 1), in agreement with previous studies [1-3,10,11].

**Figure 2:**
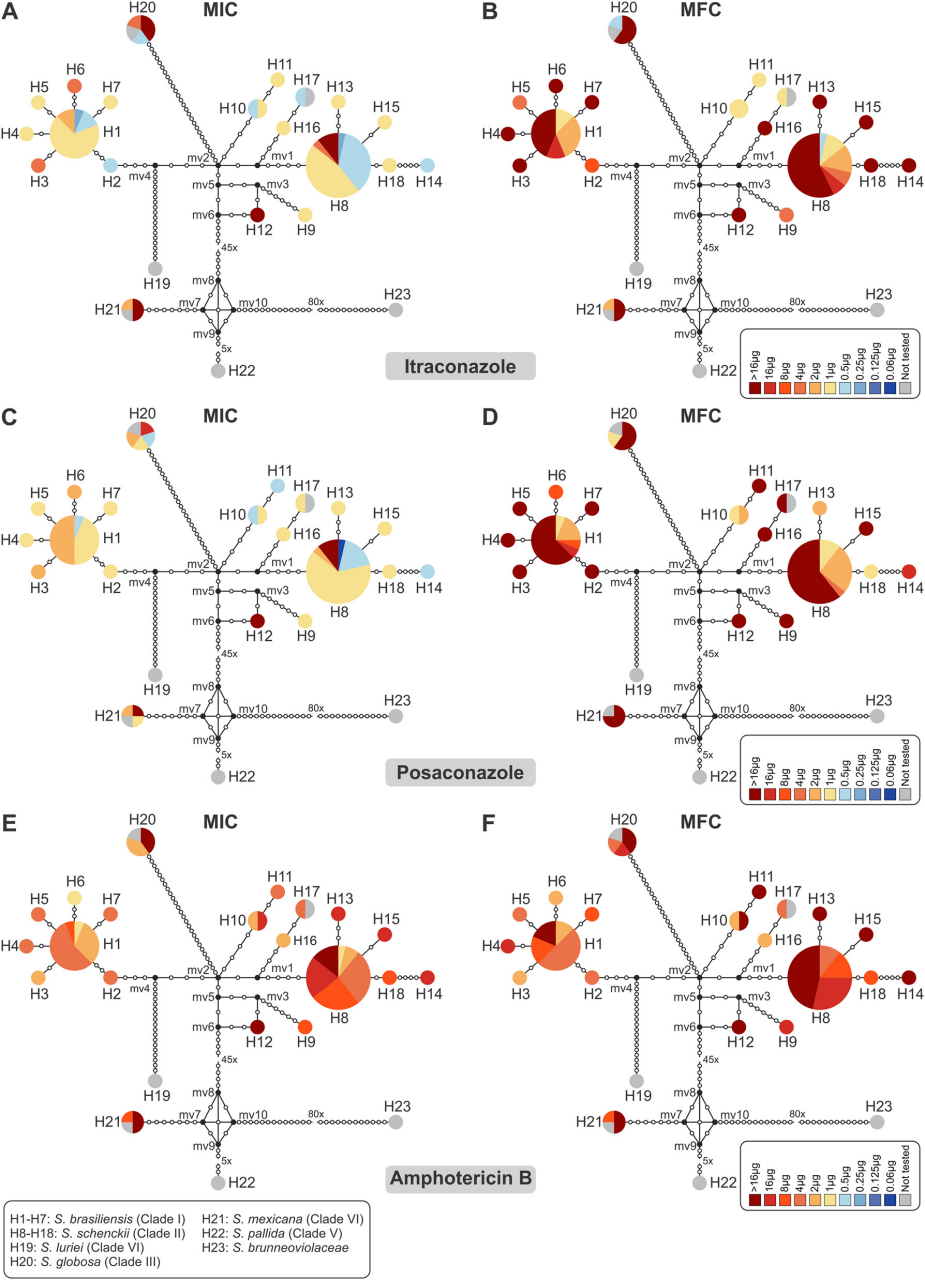
**Genetic diversity and antifungal susceptibility profiles in causative agents of sporotrichosis.** Median-joining haplotype network of *Sporothrix schenckii* complex isolates based on calmodulin and ITS concatenated sequences. The circumference size is proportional to the haplotype frequency. Black dots (median vectors) represent unsampled or extinct haplotypes in the population. Mutational steps are represented by white dots and, in cases of long branches, by values. The charts represent the MIC **(A, C, and E)** and MFC **(B, D, and F)** distributions within the population for ITC, PCZ, and AMB.

Table 1 summarizes the MIC and MFC ranges for the human clinical *Sporothrix* isolates. *Sporothrix brasiliensis* exhibited low genetic diversity (π = 0.00194), and comprised seven haplotypes (H1–H7). Of the tested drugs, the triazoles ITC and PCZ showed the best activity against *S. brasiliensis* strains, with MICs ranging from 0.25 to 4 μg/mL (Figure 2A) and from 0.5 to 2 μg/mL (Figure 2C), respectively. The MICs for ITC were lower in *S. brasiliensis* compared to in *S. schenckii* (*p* = 0.0001) and *S. mexicana* (*p* = 0.0031). We detected moderate activity for the polyene AMB against *S. brasiliensis* strains, with MICs ranging from 1 to 8 μg/mL (Figure 2E), while CAS, 5FC, FLC, and VRC showed poor activity.

**Table 1:** **Minimum inhibitory concentration (MIC) and minimum fungicidal concentration (MFC) of clinical isolates belonging to the *Sporothrix schenckii *** **complex**

**Species**		**AMB (µg/mL)**		**FLC (µg/mL)**		**ITC (µg/mL)**		**VRC (µg/mL)**		**PCZ (µg/mL)**		**5FC (µg/mL)**		**CAS (µg/mL)**	
		**MIC**	**MFC**	**MIC**	**MFC**	**MIC**	**MFC**	**MIC**	**MFC**	**MIC**	**MFC**	**MIC**	**MFC**	**MIC**	**MFC**
*S. brasiliensis* (n = 22)	Range	1-8	2 - >16	>64	>64	0.25 - 4	1 - > 16	2 - >16	16 - >16	0.5 - 2	1 - >16	64 - >64	>64	>16	>16
	50%	4	4	>64	>64	1	16	16	>16	1	>16	>64	>64	>16	>16
	90%	4	16	>64	>64	2	>16	>16	>16	2	>16	>64	>64	>16	>16
*S. schenckii* (n = 39)	Range	1 - >16	2 - >16	>64	>64	0.25 - >16	0.5 - > 16	4 - >16	4 - >16	0.06 - >16	1 - >16	64 - >64	64 - >64	2 - >16	>16
	50%	8	16	>64	>64	1	16	16	>16	1	>16	64	>64	>16	>16
	90%	>16	>16	>64	>64	4	>16	>16	>16	2	>16	>64	>64	>16	>16
*S. globosa* (n = 4)	Range	2 - >16	4 - >16	>64	>64	0.5 - >16	0.5 - >16	2 - 8	8 - >16	0.5 - 16	1 - >16	8 - >64	>64	8 - > 16	>16
	50%	-	-	-	-	-	-	-	-	-	-	-	-	-	-
	90%	-	-	-	-	-	-	-	-	-	-	-	-	-	-
*S. mexicana* (n = 3)	Range	8 - >16	8 - >16	>64	>64	2 - >16	2 - >16	1 - >16	16 - > 16	1 - >16	>16	64 - >64	>64	8 - >16	>16
	50%	-	-	-	-	-	-	-	-	-	-	-	-	-	-
	90%	-	-	-	-	-	-	-	-	-	-	-	-	-	-
Overall (n = 68)	Range	1 - >16	2 - >16	>64	>64	0.25 - >16	0.5 - >16	1 - >16	4 - >16	0.06 - >16	1 - >16	8 - >64	64 - >64	2 - >16	>16
	50%	4	16	>64	>64	1	>16	16	>16	1	>16	>64	>64	>16	>16
	90%	>16	>16	>64	>64	>16	>16	>16	>16	2	>16	>64	>64	>16	>16

*Sporothrix schenckii* represented by the genetically heterogeneous clade II (π = 0.00917) was divided into ten haplotypes (H8–H18) and showed a broad MIC range for the different antifungals. ITC (Figure 2A) and PCZ (Figure 2C) presented good activity against most *S. schenckii s. str.* strains, with MICs ranging from 0.25 to >16 μg/mL and from 0.06 to >16 μg/mL, respectively. The MICs for PCZ were slightly higher in *S. schenckii* compared to in *S. brasiliensis* (*p* = 0.0076). AMB presented high MICs against *S. schenckii* isolates, ranging from 1 to >16 μg/mL (Figure 2E), while the remaining compounds—CAS, 5FC, FLC, and VRC—showed very poor activities. Each of the evaluated drugs demonstrated poor *in vitro* efficacy against the tested *S. globosa* (H20) and *S. mexicana* (H21) strains (Figure 2).

As expected, MFCs were at least two-fold higher than the MICs for each drug (Figure 2B, D and F). AMB presented lower MFC values compared to those obtained for the other tested drugs (*p* = 0.0011; Figure 2F). The MFC for AMB was also lower among *S. brasiliensis* isolates compared to in *S. schenckii* (*p* = 0.0001) and *S. mexicana* (*p* = 0.0361). Evaluating the MIC and MFC values together, we identified several multidrug resistant isolates among the *S. schenckii s. str.* strains. In particular, the deviating haplotype 12 (H12; Figure 2B, D and F), which was represented by a Brazilian strain of human origin (Ss51), showed cross-resistance to all azole drugs and polyenes.

## Discussion

Given the high intrinsic antifungal resistance in *Sporothrix*, studies correlating antifungal susceptibility and genetic diversity among etiological agents of sporotrichosis are overdue. Here we used haplotype networks to estimate genetic diversity among clinical *Sporothrix* species. This approach has the strength of enabling the recognition of genetically deviating strains in a population, rather than randomly sampled frequent haplotypes with similar genetic background, as represented by the frequent haplotypes H1 and H8 in the *S. brasiliensis* and *S. schenckii* populations, respectively. However, haplotype-based strategies are dependent on the molecular marker used to estimate diversity, the set of strains comprising the database as well as the network methods [31]. To date, the partial sequence of *CAL* and ITS region (ITS1/2 + 5.8 s) has been used to estimate genetic diversity and taxonomy of clinical *Sporothrix* species [2,5,10,11]. Indeed, due to its highly discriminatory power and polymorphisms we choose these regions to give insights into the genetic diversity in sympatric populations of *S. brasiliensis* and *S. schenckii s. str*.

A genetically homogeneous population may be expected to have similar susceptibility profiles among the individuals, but increased fitness during population expansion can result in a diversity of susceptibility profiles. Alternatively, differential responses may emerge over time in ancestral genetically diverse species. Of the phylogenetically related species *S. brasiliensis* and *S. schenckii s. str.,* the former has a lower genetic diversity [2,3,10,13], and here we found that it also had a correspondingly lower variability of *in vitro* susceptibility. A larger degree of genetic diversity has been observed among *S. schenckii s. str.* isolates, which is accompanied by greater variation in antifungal profiles [14] virulence [12] and genomic organization [13]. Moreover, the presence of median vectors (mv1-3, 5, 6) in the haplotype network (Figure 2) may indicate extinct or intermediate unsampled haplotypes within the *S. schenckii s. str.* populations, possibly suggesting the existence of even more variation. Conversely, the absence of median vectors in the *S. brasiliensis* population strongly suggests that our analysis detected most of the strain diversity circulating in the human epidemic, thus supporting the low variation of the MIC and MFC values in this species.

Itraconazole is the drug of choice for treating endemic mycoses caused by thermodimorphic fungi, including sporotrichosis. Our findings regarding the *in vitro* activities of the main antifungal drugs are in agreement with previous studies, indicating that ITC and PCZ were moderately effective against isolates of *S. brasiliensis* and *S. schenckii s. str. *[14,32]. Despite the relatively high values found in all studies, several authors have demonstrated the efficacy of ITC in clinical outcome. Barros *et al. *[17] studied the clinical treatment of a large number of patients in Rio de Janeiro, and reported the success of ITC treatment (50–400 mg/day) in 94.6% patients (n = 610). Although this study did not molecularly identify the phylogenetic species involved, it was most likely *S. brasiliensis*, as this species is highly prevalent in Rio de Janeiro due to feline sporotrichosis outbreaks [1-3,10]. Such an epidemiological profile could explain the favorable results for ITC. The relatively low MICs found for PCZ also suggest this molecule to be a promising drug in the treatment of sporotrichosis caused by *S. schenckii s. str*. or *S. brasiliensis*. Additionally, PCZ also showed low MICs against 2 out of 3 clinical isolates of the rare species *S. mexicana*, including CBS 132928 (MIC = 1 μg/mL) and CBS 132927 (MIC = 2 μg/mL). Only a few strains of *S. mexicana* have been described in the literature [1,10], and the species has been reported as tolerant to most commercially available drugs [14,33].

Resistance to amphotericin B has been reported in emerging pathogens, such as *Aspergillus terreus*, *Candida lusitaniae*, *Fusarium* spp., *Scedosporium prolificans*, and *Trichosporon asahii *[34,35]. Here we found high MICs for AMB in five isolates of *S. schenckii s. str.* (Ss17, Ss22, Ss110, and Ss119 from H8, and isolate Ss51 from H12) with MIC and MFC values of >16 μg/mL. The multidrug resistant phenotype found for isolate Ss51 (H12; Figure 2B, D, and F) is particularly noteworthy in relation to its clinical origin, where it showed moderate virulence in a murine model [12]. The high MICs found for the remaining compounds (FLC, VRC, CAS, and 5FC) are in agreement with previous reports [14,32,36].

Fungistatic drugs are capable of inhibiting the cell growth and reproduction of fungi without destroying them. Drug-sensitive pathogens may evolve resistance under the selective pressure imposed by continuous exposure to fungistatic drugs [37]. The molecular mechanism that lies behind the recent emergence of drug-resistant phenotype among *Sporothrix* species is currently unknown. However, judging from other fungi, the increased and prolonged use of triazoles has raised concerns about resistant infections by *Cryptococcus neoformans *[38], *Candida albicans *[39] and *Aspergillus fumigatus *[40]. Azole resistance may occur through a diversity of mechanisms including the upregulation of multidrug transporter genes that leads to enhanced efflux of azoles and therefore reduce drug accumulation [41]; multiple genetic alterations of the target enzyme that can affect the affinity of the enzyme and therefore prevents azole binding [42], and alteration of metabolism, usually sterol synthesis [43]. More recently, genetic studies correlating antifungal resistance and alteration in chromosome copy number and genomics architecture has delivered an exciting view on the molecular mechanisms governing the increased fitness to the phenotype of azole-resistance [38,44-46].

*Candida albicans *[39,44,45] and *Cryptococcus neoformans *[38,46,47] triazole-resistant isolates very often contain an abnormal number of chromosomes. Such genomic plasticity may offer additional copies of drug resistance genes leading chromosomal aneuploidies isolates to overcoming the drug effects [44,45] and allowing rapid adaptive evolution [47]. Although this phenomenon may occur spontaneously [47], drug-resistant aneuploidies arise very frequently within drug-exposed fungal populations and this may support our recent findings on chromosomal polymorphisms in *Sporothrix* species [13]. The intra-specific polymorphisms in chromosome number and size in *S. schenckii s. str.* suggested that aneuploidy occur among clinical isolates, and it is tempting to hypothesize that this phenomenon could, in part, be responsible for the differences in drug profiles observed here. Therefore, the high karyotype diversity observed for *S. schenckii s. str. *[13] is reflected in the genetic diversity found in our haplotype network (Figure 2). However, testing for this pattern requires a larger number of isolates with dissimilar antifungal profiles as well as chromosomal polymorphism.

Fungicidal drugs may be defined as those that lead to a reduction of 99.9% of the initial inocula [48]. It is difficult to make comparisons between published MFCs studies, since most were performed before the introduction of clinical species beyond *S. schenckii s. str.* to the genus *Sporothrix*. Two or more of these newer species were likely involved in the studies of McGinnis *et al. *[49] and Silveira *et al. *[50]. In all clinical *Sporothrix* spp. evaluated, we found significant differences between the minimum concentrations needed to inhibit fungal cell growth and the concentration required for colony count reduction. In all studied species, most isolates were able to grow after 72 h in the presence of each drug at the maximum concentration tested, which is in agreement with results obtained by Trilles *et al. *[36]. The moderately low MICs for triazoles imply a great resistance to killing among isolates of *S. brasiliensis*, the most pathogenic among *Sporothrix* species.

Spontaneous cure, as well as relapse, are common features of sporotrichosis. Our present data show that most antifungal agents had only a fungistatic effect against clinical isolates. It remains unclear whether there is a correlation between *in vitro* MIC/MFC data and clinical outcome in human sporotrichosis. Experimental murine models have shown the efficacy of posaconazole (5 mg/kg) for treating *S. brasiliensis* and *S. schenckii s. str.* infections [51]. Voriconazole (40 mg/kg) has been demonstrated to only reduce fungal load in mice infected with *S. schenckii s. str.*, and to have no activity against *S. brasiliensis *[52]. No studies to date have linked the dissimilar phylogenetic species to *in vitro* and *in vivo* data. Our *in vitro* results for the highly tolerant strain CBS 133020 (=Ss265, haplotype 7), an isolate originated from a disseminated case of *S. brasiliensis* in an HIV patient [9], shown that despite ITC and AMB MICs of 1 and 4 μg/mL, respectively, and MFCs of >16 and 8 μg/mL, respectively, the patient showed positive clinical outcome after administration of intravenous AMB (including 10 days on L-AMB) [9], indicating a need to monitor MFCs values before and during treatment.

## Conclusions

Our study presents antifungal susceptibility profiles using MIC and MFC data, which is particularly noteworthy in light of recent taxonomic changes in medically relevant *Sporothrix* species. We assessed a genetically diverse set of strains with varying degrees of disease severity (fixed cutaneous, lymphocutaneous, or disseminated), and the antifungal susceptibility assays revealed a broad spectrum for the triazoles (ITC, PCZ, and VRC). This finding may have implications for the choice of antifungal therapy in different epidemiological risk groups, potentially having a substantial impact on clinical outcome. Furthermore, our data indicate the existence of multidrug resistant strains within the *S. schenckii* complex, underlining the need for continuous susceptibility screening. Further studies should be conducted to connect *in vitro* and clinical data, as well as to investigate the molecular mechanisms underlying the different resistance profiles among *S. schenckii* and its relatives.

## Abbreviations

ITS: Internal transcribed spacer; CAL: Calmodulin; MICs: Minimum inhibitory concentration; MFC: Minimum fungicidal concentration; AMB: Amphotericin B; FLC: Fluconazole; ITC: Itraconazole; VRC: Voriconazole; PCZ: Posaconazole; 5FC: Flucytosine; CAS: Caspofungin; s. str.: *sensu stricto*; s.l.: *sensu lato*; ML: Maximum likelihood; NJ: Neighbor-joining; mv: Median vector.

## Competing interests

The authors declare that they have no competing interests. The authors alone are responsible for the content and writing of the paper.

## Authors’ contributions

AMR and DCP performed the experiments. AMR, ALC, and ZPC designed the experiments. AMR, GSdH, RSNB, JJCS, MFG, ALC, and ZPC analyzed the data, and drafted the manuscript together. All authors read and approved the final manuscript.

## Pre-publication history

The pre-publication history for this paper can be accessed here:

http://www.biomedcentral.com/1471-2334/14/219/prepub

## Supplementary Material

Additional file 1: Table S1 Strains, species, origin, haplotypes, and GenBank accession numbers (*CAL* and ITS) for the *Sporothrix* spp. isolates used in this study and the reference strains. Click here for file
