# Supplementary material for: Genetic diversity and antifungal susceptibility profiles in causative agents of sporotrichosis
Source: BMC Infect Dis. 2014 Apr 23;14:219. doi: 10.1186/1471-2334-14-219 (PMC4021050; doi:10.1186/1471-2334-14-219)
Supplement: Additional file 1: Table S1 — Strains, species, origin, haplotypes, and GenBank accession numbers (CAL and ITS) for the Sporothrix spp. isolates used in this study and the reference strains. [file 1471-2334-14-219-S1.docx]

**Supplementary Data**

**Supplementary Table 1.** Strains, species, origin, haplotypes and GenBank accession numbers (*CAL* and ITS) for the *Sporothrix* spp. isolates used in this study and the reference strains.

| **Isolate** | **Other Code** | **Species** | **Source** | **Country** | ***CAL*+ITS Haplotype** | **CAL** | **ITS** | **Reference** |
| --- | --- | --- | --- | --- | --- | --- | --- | --- |
| Ss05 | CBS 132985 | *Sporothrix brasiliensis* | Feline | MG, Brazil | Hap 01 | KC693830 | KF961142 | [1,2] |
| Ss07 | CBS 132986 | *Sporothrix brasiliensis* | Human | MG, Brazil | Hap 01 | KC693831 | KF961143 | [1] |
| Ss12 | - | *Sporothrix brasiliensis* | Human | MG, Brazil | Hap 01 | KC693835 | KF961144 | [1,2] |
| Ss14 | - | *Sporothrix brasiliensis* | Human | MG, Brazil | Hap 01 | KF943632 | KF961145 | This study |
| Ss33 | - | *Sporothrix brasiliensis* | Human | PR, Brazil | Hap 01 | KF943637 | KF961146 | This study |
| Ss34 | - | *Sporothrix brasiliensis* | Human | PR, Brazil | Hap 02 | KF943638 | KF961147 | This study |
| Ss37 | - | *Sporothrix brasiliensis* | Human | PR, Brazil | Hap 01 | KF943639 | KF961148 | This study |
| Ss38 | - | *Sporothrix brasiliensis* | Human | PR, Brazil | Hap 01 | KC693844 | KF961149 | [1,2] |
| Ss43 | - | *Sporothrix brasiliensis* | Human | CE, Brazil | Hap 03 | JX077112 | KF961150 | This study |
| Ss44 | - | *Sporothrix brasiliensis* | Human | CE, Brazil | Hap 01 | KF943641 | KF961151 | This study |
| Ss52 | - | *Sporothrix brasiliensis* | Human | SP, Brazil | Hap 04 | KC693845 | KF574444 | [1-3] |
| Ss54 | CBS 132990 | *Sporothrix brasiliensis* | Feline | RS, Brazil | Hap 01 | JQ041903 | JN885580 | [1-4] |
| Ss55 | - | *Sporothrix brasiliensis* | Human | RS, Brazil | Hap 01 | KC693847 | KF961152 | [1,2] |
| Ss56 | - | *Sporothrix brasiliensis* | Human | RS, Brazil | Hap 01 | KC693848 | KF961153 | [1] |
| Ss57 | - | *Sporothrix brasiliensis* | Human | RS, Brazil | Hap 01 | KF943645 | KF961154 | This study |
| Ss62 | CBS 132991 | *Sporothrix brasiliensis* | Human | ES, Brazil | Hap 05 | JX077113 | KF961120 | [4] |
| Ss99 | - | *Sporothrix brasiliensis* | Human | RJ, Brazil | Hap 01 | KF574460 | KF574442 | [3] |
| Ss104 | - | *Sporothrix brasiliensis* | Human | MT, Brazil | Hap 01 | KF574461 | KF574443 | [3] |
| **Isolate** | **Other Code** | **Species** | **Source** | **Country** | **CAL+ITS Haplotype** | **CAL** | **ITS** | **Reference** |
| Ss128 | - | *Sporothrix brasiliensis* | Human | SP, Brazil | Hap 06 | KC693861 | KF961155 | [1] |
| FMR 8314 | IPEC 16919 | *Sporothrix brasiliensis* | Human | RJ, Brazil | Hap 01 | AM116899 | KF574441 | [5,6] |
| CBS 120339^T^ | IPEC 16490 | *Sporothrix brasiliensis* | Human | RJ, Brazil | Hap 01 | AM116898 | KF574440 | [5,6] |
| Ss265 | CBS 133020 | *Sporothrix brasiliensis* | Human | MG, Brazil | Hap 07 | JN204360 | KF574445 | [3,7] |
| Ss03 | CBS 132963 | *Sporothrix schenckii* | Human | RS, Brazil | Hap 08 | JX077117 | KF574446 | [2-4] |
| Ss04 | - | *Sporothrix schenckii* | Human | RS, Brazil | Hap 08 | JX077118 | KF961156 | [2,4] |
| Ss13 | - | *Sporothrix schenckii* | Human | MG, Brazil | Hap 08 | KC693836 | KF961157 | [1,2] |
| Ss15 | - | *Sporothrix schenckii* | Human | MG, Brazil | Hap 08 | KC693837 | KF961158 | [1] |
| Ss16 | - | *Sporothrix schenckii* | Human | PI, Brazil | Hap 09 | JQ041898 | KF961159 | [8] |
| Ss17 | - | *Sporothrix schenckii* | Human | PR, Brazil | Hap 08 | KC693838 | KF961160 | [1] |
| Ss19 | - | *Sporothrix schenckii* | Human | PR, Brazil | Hap 08 | KF943633 | KF961161 | This study |
| Ss22 | CBS 132964 | *Sporothrix schenckii* | Human | PR, Brazil | Hap 08 | KF943634 | KF961162 | This study |
| Ss36 | - | *Sporothrix schenckii* | Human | PR, Brazil | Hap 08 | KC693843 | KF961163 | [1] |
| Ss40 | - | *Sporothrix schenckii* | Human | CE, Brazil | Hap 08 | JQ041900 | JN885577 | [3,8] |
| Ss42 | CBS 132966 | *Sporothrix schenckii* | Human | CE, Brazil | Hap 08 | KF943640 | KF961164 | This study |
| Ss45 | - | *Sporothrix schenckii* | Human | GO, Brazil | Hap 10 | KJ020358 | KF961165 | This study |
| Ss46 | - | *Sporothrix schenckii* | Human | GO, Brazil | Hap 10 | KF943642 | KF961166 | This study |
| Ss48 | - | *Sporothrix schenckii* | Human | GO, Brazil | Hap 11 | KF943643 | KF961167 | This study |
| Ss51 | - | *Sporothrix schenckii* | Human | PA, Brazil | Hap 12 | JQ041902 | JN885579 | [3,8] |
| Ss59 | - | *Sporothrix schenckii* | Human | SP, Brazil | Hap 08 | KF943647 | KF961168 | This study |
| Ss61 | - | *Sporothrix schenckii* | Soil | SP, Brazil | Hap 08 | KF561244 | KF574447 | [3] |
| Ss63 | CBS 132968 | *Sporothrix schenckii* | Human | ES, Brazil | Hap 08 | JX077123 | KF961121 | [4] |
| Ss64 | - | *Sporothrix schenckii* | Human | ES, Brazil | Hap 08 | JX077124 | KF961169 | [4] |
| **Isolate** | **Other Code** | **Species** | **Source** | **Country** | **CAL+ITS Haplotype** | **CAL** | **ITS** | **Reference** |
| Ss73 | - | *Sporothrix schenckii* | Human | RJ, Brazil | Hap 08 | KC693853 | KF961170 | [1,2] |
| Ss75 | - | *Sporothrix schenckii* | Human | RJ, Brazil | Hap 13 | KC693854 | KF961123 | [1] |
| Ss78 | - | *Sporothrix schenckii* | Human | RJ, Brazil | Hap 08 | KC693855 | KF961171 | [1,2] |
| Ss102 | CBS 132970 | *Sporothrix schenckii* | Human | SP, Brazil | Hap 08 | KF943671 | KF961172 | This study |
| Ss110 | - | *Sporothrix schenckii* | Human | MG, Brazil | Hap 08 | KF943677 | KF961173 | This study |
| Ss114 | - | *Sporothrix schenckii* | Human | SP, Brazil | Hap 14 | KJ020357 | KJ020360 | This study |
| Ss116 | - | *Sporothrix schenckii* | Human | SP, Brazil | Hap 08 | KF943680 | KF961131 | This study |
| Ss118 | CBS 132974 | *Sporothrix schenckii* | Human | SP, Brazil | Hap 15 | JX077126 | KF961174 | [2,4] |
| Ss119 | - | *Sporothrix schenckii* | Human | SP, Brazil | Hap 08 | KF943682 | KF961175 | This study |
| Ss124 | - | *Sporothrix schenckii* | Human | SP, Brazil | Hap 08 | KF943687 | KF961176 | This study |
| Ss126 | - | *Sporothrix schenckii* | Human | SP, Brazil | Hap 08 | JQ041904 | JN885581 | [2,3,8] |
| Ss130 | - | *Sporothrix schenckii* | Human | PE, Brazil | Hap 16 | KF943690 | KF961135 | This study |
| Ss136 | - | *Sporothrix schenckii* | Human | PE, Brazil | Hap 08 | KF943692 | KF961177 | This study |
| Ss137 | - | *Sporothrix schenckii* | Human | PE, Brazil | Hap 08 | KF574462 | KF574448 | This study |
| Ss138 | - | *Sporothrix schenckii* | Human | PB, Brazil | Hap 08 | KF943693 | KF961136 | This study |
| Ss141 | CBS 132975 | *Sporothrix schenckii* | Human | GO, Brazil | Hap 08 | JQ041905 | JN885582 | [3,8] |
| Ss143 | - | *Sporothrix schenckii* | Human | PA, Brazil | Hap 17 | JQ041906 | JN885583 | [3,8] |
| Ss144 | - | *Sporothrix schenckii* | Human | RS, Brazil | Hap 18 | KF943695 | KF961178 | This study |
| Ss158 | - | *Sporothrix schenckii* | Human | AM, Brazil | Hap 08 | KF943698 | KF961137 | This study |
| Ss243 | - | *Sporothrix schenckii* | Human | SP, Brazil | Hap 08 | KJ020359 | KF961179 | This study |
| CBS 359.36^T^ | - | *Sporothrix schenckii* | Human | USA | Hap 17 | AM117437 | FJ545232 | [5,6] |
| CBS 937.72^T^ | ATCC 18616 | *Sporothrix luriei* | Human | South Africa | Hap 19 | AM747302 | AB128012 | [9,10] |
| Ss06 | CBS 132922 | *Sporothrix globosa* | Human | MG, Brazil | Hap 20 | JF811336 | JN885574 | [1,2,4] |
| **Isolate** | **Other Code** | **Species** | **Source** | **Country** | **CAL+ITS Haplotype** | **CAL** | **ITS** | **Reference** |
| Ss41 | CBS 132923 | *Sporothrix globosa* | Human | CE, Brazil | Hap 20 | JF811337 | KF574456 | [1,2,4] |
| Ss49 | CBS 132924 | *Sporothrix globosa* | Human | GO, Brazil | Hap 20 | JF811338 | KF961180 | [1,2,4] |
| Ss236 | CBS 132925 | *Sporothrix globosa* | Human | MG, Brazil | Hap 20 | KC693877 | KF961181 | [1,2] |
| CBS 120340^T^ | FMR 8600 | *Sporothrix globosa* | Human | Spain | Hap 20 | AM116908 | FN549905 | [5,6] |
| Ss131 | CBS 132926 | *Sporothrix mexicana* | Human | PE, Brazil | - | JF811339 | - | [4] |
| Ss132 | CBS 132927 | *Sporothrix mexicana* | Human | SP, Brazil | Hap 21 | JF811340 | KF574457 | [1,2,4] |
| Ss133 | CBS 132928 | *Sporothrix mexicana* | Human | PE, Brazil | Hap 21 | JF811341 | KF961182 | [1,2,4] |
| CBS 120341^T^ | FMR 9108 | *Sporothrix mexicana* | Vegetal | Mexico | Hap 21 | AM398393 | FN549906 | [5,6] |
| Ss327 | PG3 | *Sporothrix pallida* | Feline | RS, Brazil | Hap 22 | KF574471 | KF574458 | This study |
| CBS 124561^T^ | FMR 9338 | *S. brunneoviolaceae* | Soil | Spain | Hap 23 | KF574472 | FN546959 | [10] |

IPEC, Instituto de Pesquisa Clínica Evandro Chagas, Fiocruz, Brazil; FMR, Facultat de Medicina i Ciències de la Salut, Reus, Spain; CBS, Centraalbureau voor Schimmelcultures, Utrecht, The Netherlands; ATCC, American Type Culture Collection, Manassas, VA, USA; ^T^, type strain. All “Ss” strains belong to the culture collection of the Federal University of São Paulo (UNIFESP). AM, Amazonas; CE, Ceará; ES, Espírito Santo; GO, Goiás; MG, Minas Gerais; MT, Mato Grosso; PA, Pará; PB, Paraíba; PE, Pernambuco; PI, Piauí; PR, Paraná; RJ, Rio de Janeiro; RS, Rio Grande do Sul; SP, São Paulo.

**Supplementary References:**

1. Rodrigues AM, de Melo Teixeira M, de Hoog GS, Schubach TMP, Pereira SA, Fernandes GF, Bezerra LML, Felipe MS, de Camargo ZP: **Phylogenetic analysis reveals a high prevalence of *Sporothrix brasiliensis* in feline sporotrichosis outbreaks**. *PLoS Negl Trop Dis* 2013, **7**(6):e2281.

2. Rodrigues AM, de Hoog GS, Camargo ZP: **Genotyping species of the *Sporothrix schenckii* complex by PCR-RFLP of calmodulin.** *Diagn Microbiol Infect Dis* 2014, 78(4):383–387.

3. Sasaki AA, Fernandes GF, Rodrigues AM, Lima FM, Marini MM, dos S. Feitosa L, de Melo Teixeira M, Felipe MSS, da Silveira JF, de Camargo ZP: **Chromosomal polymorphism in the *Sporothrix schenckii* complex.** *PLoS ONE* 2014, 9(1):e86819.

4. Rodrigues AM, de Hoog S, de Camargo ZP: **Emergence of pathogenicity in the *Sporothrix schenckii* complex**. *Med Mycol* 2013, **51**(4):405–412.

5. Marimon R, Cano J, Gené J, Sutton DA, Kawasaki M, Guarro J: ***Sporothrix brasiliensis*, *S. globosa*, and *S. mexicana*, three new *Sporothrix* species of clinical interest**. *J Clin Microbiol* 2007, **45**(10):3198-3206.

6. Marimon R, Gené J, Cano J, Trilles L, Dos Santos Lazéra M, Guarro J: **Molecular phylogeny of *Sporothrix schenckii***. *J Clin Microbiol* 2006, **44**(9):3251-3256.

7. Silva-Vergara ML, de Camargo ZP, Silva PF, Abdalla MR, Sgarbieri RN, Rodrigues AM, dos Santos KC, Barata CH, Ferreira-Paim K: **Disseminated *Sporothrix brasiliensis* infection with endocardial and ocular involvement in an HIV-infected patient**. *Am J Trop Med Hyg* 2012, **86**(3):477-480.

8. Fernandes GF, dos Santos PO, Rodrigues AM, Sasaki AA, Burger E, de Camargo ZP: **Characterization of virulence profile, protein secretion and immunogenicity of different *Sporothrix schenckii sensu stricto* isolates compared with *S. globosa* and *S. brasiliensis* species**. *Virulence* 2013, **4**(3):241-249.

9. Marimon R, Gené J, Cano J, Guarro J: ***Sporothrix luriei*: a rare fungus from clinical origin**. *Med Mycol* 2008, **46**(6):621-625.

10. Madrid H, Gené J, Cano J, Silvera C, Guarro J: ***Sporothrix brunneoviolacea* and *Sporothrix dimorphospora*, two new members of the *Ophiostoma stenoceras*-*Sporothrix schenckii* complex**. *Mycologia* 2010, **102**(5):1193-1203.
